# A machine learning workflow for raw food spectroscopic classification in a future industry

**DOI:** 10.1038/s41598-020-68156-2

**Published:** 2020-07-08

**Authors:** Panagiotis Tsakanikas, Apostolos Karnavas, Efstathios Z. Panagou, George-John Nychas

**Affiliations:** School of Food and Nutritional Sciences, Department of Food Science and Human Nutrition, Laboratory of Microbiology and Biotechnology of Foods, Agricultural University of Athens, Iera Odos 75, 11855 Athens, Greece

**Keywords:** Surface spectroscopy, Computational science

## Abstract

Over the years, technology has changed the way we produce and have access to our food through the development of applications, robotics, data analysis, and processing techniques. The implementation of these approaches by the food industry ensure quality and affordability, reducing at the same time the costs of keeping the food fresh and increase productivity. A system, as the one presented herein, for raw food categorization is needed in future food industries to automate food classification according to type, the process of algorithm approaches that will be applied to every different food origin and also for serving disabled people. The purpose of this work was to develop a machine learning workflow based on supervised PLS regression and SVM classification, towards automated raw food categorization from FTIR. The system exhibited high efficiency in multi-class classification of 7 different types of raw food. The selected food samples, were diverse in terms of storage conditions (temperature, storage time and packaging), while the variability within each food was also taken into account by several different batches; leading in a classifier able to embed this variation towards increased robustness and efficiency, ready for real life applications targeting to the digital transformation of the food industry.

## Introduction

At the dawn of the twenty-first century, the agri-food sector is facing major challenges: first, providing the world’s population with enough to eat (Food Security)^1^ and second, ensuring that this food is safe to eat (Food Safety)^1^, while maintaining a production process within environmental constraints. These objectives have to be realized in the context of tremendous technological change, a growing lack of natural resources, and a continuous evolution of consumers’ life-styles and consumption habits, across the globe^1,2^. The food industry is obliged to operate under seemingly contradictory expectations, i.e. consumers prefer foods that are (i) convenient and fresh (minimally-processed and packaged); (ii) all “natural”—with no preservatives; (iii) potentially healthy without adverse health effects (i.e., low in fat, salt, and sugar); and (iv) produced in an environmentally sustainable manner.

Regarding these issues, the Joint Research Centre (JRC) Science for policy report^3^ investigated 4 scenarios on the identification of future challenges in the global food system and indicated the need to increase dependence on Information and Communications Technologies (ICT) to ensure traceability in the food chain and the possibility of temporary failure or fraud and terrorism.

To implement this need, smart sensors have been designed to bridge the gap between appropriate food information and consumer’s needs. Similarly, the importance of ICT has been recognized as a mean to enhance the operational efficiency and productivity in the agricultural sector/food industry in the context of the Implementation Action Plan proposed by European Technology Platforms (ETPs), which are industry-led stakeholder fora, recognized by the European Commission as key actors in driving innovation, knowledge transfer and European competitiveness^4^. The use of sensors is of vital importance in the food industry; their potential of taking non-invasive measurements on, in or at line without destructing the food product is a prerequisite for the food industry of the future^5^.

Nowadays many different sensors (e.g. NIR, FTIR, RAMAN, Multi or High Spectral Imaging—surface chemistry) have been employed by the food sector to evaluate freshness, microbial quality, adulteration, food origin, etc.^6–10^. However, due to the complexity of these measurements, data analytics (DA) should be considered as an essential step, to provide solid and valid information to stakeholders^5,11,12^ with regard to the quality characteristics mentioned above. Indeed these measurements in tandem with DA have been found to tackle basic issues regarding the implementation of rapid methods in the Food sector. Although a number of studies have shown that the combination of sensors and DA^12^ can provide accurate information regarding the freshness, safety, and quality integrity of specific products, its limitation is evident since they cannot discern the product per se; thus a measurement from fish cannot be characterized as such, while if used for meat products it will fail to provide the correct answer. In other words, it is clear that in these measurements a region of the spectrum should be used as a key which can characterize the identity of the product. This key region(s) in the measurements is (are) essential and will allow the ‘classification’ of food system so as to “discriminate” different food commodities, prior to the evaluation of requested parameters such as the quality status of the product. Intuitively it can be thought as a ‘coding’ region of the data, showing the type of the food, driving and employing the use of the suitable analysis pipeline from a library of pipelines resident on a local database or on the cloud. At the same time it can be used to facilitate the life of disabled persons (e.g., the blind) or the needs of a huge futuristic megastore that imports food products from different producers and distribute these to consumers using advanced communication technologies, i.e. the Internet of Foods. The basic idea underlying the aforementioned “discrimination of different food commodities” is that the spectrum from a sensor for a specific raw material would exhibit some unique features/properties compared to the spectrum of other food systems. This has to be performed regardless of any inherent variation of the raw material per se due to other origins, e.g. storage conditions, storage time, animal diet, etc. Another possible application of the present work is the current need for food recognition prior and towards a fully automated food quality assessment system in the industry with several raw food types as raw materials. Such a system will serve as a submodule for switching/enabling the appropriate food type specific algorithm/pipeline execution. Throughout the literature on food science, it is apparent that the majority of algorithms and approaches developed for food quality assessment is very different for the different types of food, in terms of spectra type, preprocessing (data normalization, filtering etc.) and regression and/or classification approaches^12^. Thus, there is need for automated selection, e.g. recognition of the food type directly from spectra, and thus redirecting to raw food type to the most suitable quality assessment approach. Actually, this need has emerged from a running EU project in which our research group participates, named PhasmaFOOD^13^. Finally and maybe the most promising application of the presented system is the sorting of raw food materials in the food industry to prepare the production chain for the manufacturing of mixed goods and recipes. One of the most advanced AI applications in the food industry is TOMRA Sorting Food^14^, which uses sensor-based optical sorting solutions with machine learning functionalities. Herein, we reach a performance of 100% accuracy without the use of AI, but with “traditional” machine learning methods, where in addition to high accuracy it also provides inferable results.

At this point it should be underlined that the presented methodology, although closely related, has no application (at least in its current form) in adulteration detection. The datasets used are not from adulteration experimental designs and do not take into account mixed adulterated samples but only pure samples. Throughout the literature there is a lot of research^15,16^ in the subject with several instruments, including FT-IR. All the approaches for adulteration detection use data from pure and mixed samples. The corresponding chemometrics are designed to identify adulteration taking into account the information from adulterated samples. Intuitively, the algorithms detect the alterations considering the variations/alterations of the spectra while adulteration in several levels occurs, where the spectrum of each sample corresponds to the whole surface of the sample. Thus, having a dataset of only pure samples cannot support the development of a system for adulteration detection purposes. In this context, the purpose of this study was to investigate the potential use of data mining and data analysis, on data acquired by a Fourier-transform infrared spectroscopy (FT-IR) sensor, a non-destructive/non-invasive instrument to ‘identify’ food commodities from a single signal (spectrum) only.

## Results and discussion

**Figure 1:**
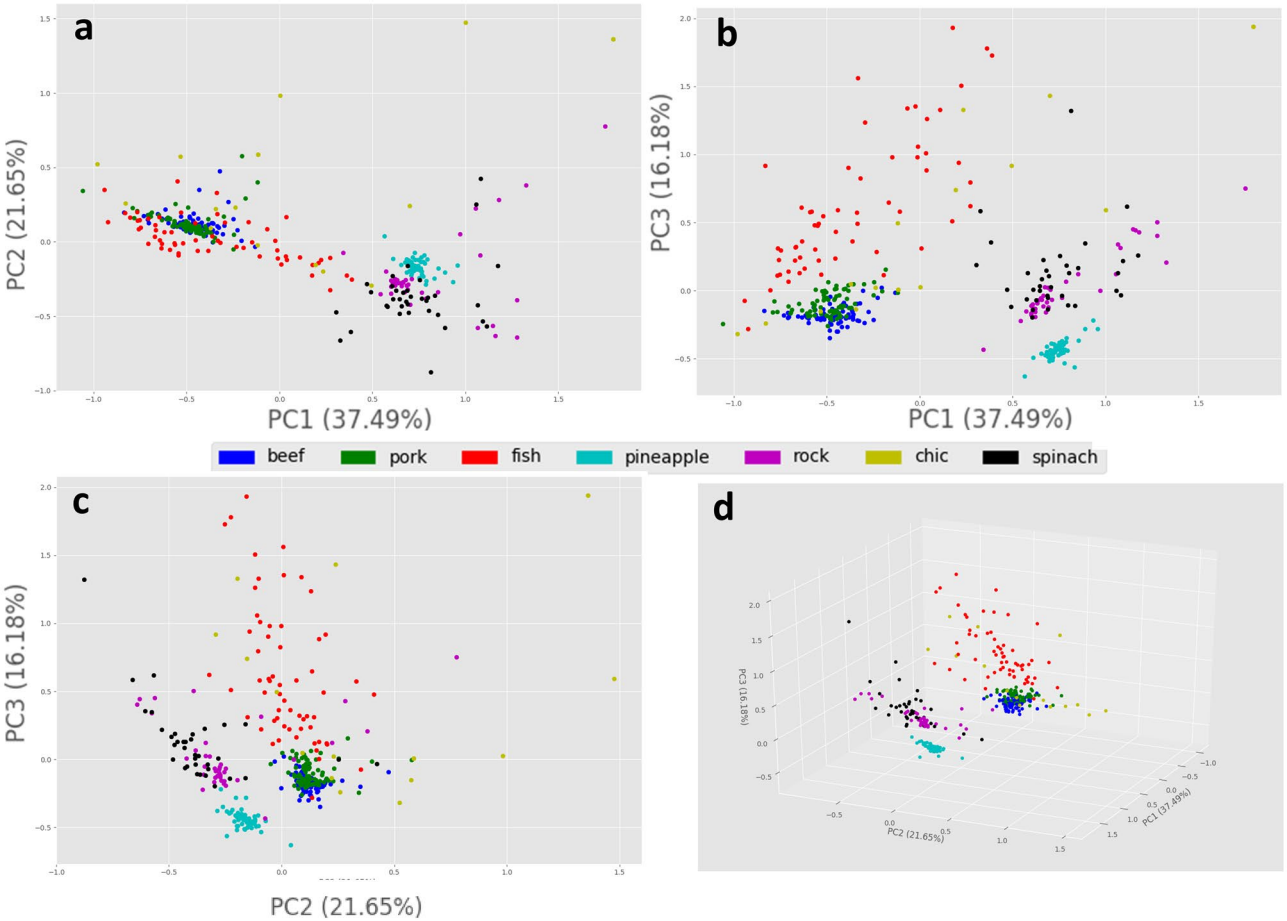
PCA plot for the three first principal components of the normalized data after feature selection via PLS regression, the 41 dimensions training dataset; (**A**) PC1-PC2 plot, (**B**) PC1-PC3 plot, (**C**) PC2–PC3 plot and (**D**) 3-D plot of the PCA.

The developed workflow for raw food material recognition employing FT-IR spectra is implemented in Python 2.7. A brief outline is provided here and more details can be found in the Methods section. First, the raw sensor data are passed via a preprocessing and normalization step Standard Normal Variate^17^ (SNV) and specifically under its robust version, RNV^18^. This step is crucial to enhance the quality of the data, remove any correlated information across the different wavelengths/wavenumbers, and also eliminate the inherent multiplicative noise. Afterward, a supervised dimensionality reduction is employed, based on Partial Least Squares Regression (PLSR)^19^. A supervised dimensionality reduction scheme has been selected to help/guide the system towards a more focused dimension estimation to the “target” of raw food classification rather than other, not relevant sample properties such as a batch of sample origin, sample storage conditions, etc. PLSR can be thought of as a feature engineering method for the following SVM classification. Finally, the classification model of the 7 raw food material types has been built using SVM classifier^20^, producing the final classification model. Figure 1 presents the data (training set) on the space of the three first principal components. The data was transformed via Principal Components Analysis and refer to the data after the normalization and feature selection by the PLS regression scheme described in Methods section. In addition, the interested reader can also refer to SI1 for the PCA and PLS plots of the data prior of the feature selection step.

The developed classification model (please refer to Methods) results to an accuracy of 100% with a lower 95% confidence interval bound at accuracy 98.5% (p-Value >> 0.0001). The accuracy corresponds to the prediction of the correct food type among the 240 independent test samples (Table 1 summarizes the confusion matrix). The significance of these results is enhanced if someone takes into account the properties of the samples consisting the data. As discussed in the Materials Section, the samples for each individual food subtype are highly variant (please refer to SI1 figures showing the mean spectrum and the standard deviation of the samples for each food type). Samples have origins from several different batches (which means inherent variance due to different sampling). Furthermore, the samples were exposed to different storage conditions in terms of time, temperature and also packaging (aerobic or modified atmosphere packaging—MAP). Table 2 presents an overview of the origin of the samples, their type and the experimental setup for which they have been acquired. It should be mentioned that as far as the storage conditions is concerned, the variance in the samples depends highly on the storage temperatures^21–23^. This is due to the different types of microorganisms^24^ (mesophiles or psychrotrophs) that can be populate and predominate on the food samples, resulting in a large variety of the byproducts they produce and thus to the chemical composition of the surface. So, it is apparent that the acquired spectra for the same food type exhibit variations, originated from the microorganisms’ byproducts; i.e. the microenvironment they create on the food. Moreover, storage time is also another significant source of variance on the data mainly due to the different levels of byproducts’ abundance^21^ and in general the physicochemical alterations of the samples (dehydration, oxidation etc.). Finally, another important source of variability of the acquired spectra of food of the same type is the packaging. Packaging, plays a major role on microorganisms’ growth on the samples^21^, since aerobic and facultative anaerobic microorganisms are prevailing one another as affected by their surroundings^25^. In conclusion, it is apparent that taking into account the different initial microbiological load along with all the aforementioned parameters (temperature, storage time and type of packaging) that affect the surface chemical composition of the samples, the acquired spectra exhibit large variations even among the same food type (please refer to SI1 to see the standard deviation for spectra of the same food type).

**Table 1:** Confusion matrix presenting the classification results.

		Predicted						
		Beef (0)	Pork (1)	Fish (2)	Pineapple (3)	Rocket (4)	Chicken (5)	Spinach (6)
Actual	Beef (0)	44	0	0	0	0	0	0
	Pork (1)	0	52	0	0	0	0	0
	Fish (2)	0	0	26	0	0	0	0
	Pineapple (3)	0	0	0	38	0	0	0
	Rocket (4)	0	0	0	0	29	0	0
	Chicken (5)	0	0	0	0	0	15	0
	Spinach (6)	0	0	0	0	0	0	36

**Table 2:** Brief description of the used samples from the specific references.

References	Food type	Case study	Experimental design
^34^	Minced beef Minced pork	Adulteration	• A two-year survey of collecting samples of minced beef and pork was conducted
^35^	Beef fillets	Spoilage	• Sterile and naturally contaminated, at 2, 8, 15 °C. Sampling along 10 days of storage
^36^	Minced pork Pork fillets Beef fillets Minced Beef	Automated image analysis—spoilage—adulteration	• Sterile pork fillets at 4° and 10 °C, aerobically and under modified atmosphere packaging • Sterile pork fillets inoculated with the specific spoilage microorganism Pseudomonas putida at 4° and 10 °C aerobically and under modified atmosphere packaging • Sterile beef fillets at 2°, 8° and 15 °C, • Naturally contaminated beef fillets, at 2°, 8° and 15 °C • Naturally contaminated beef fillets inoculated with different inocula of Salmonella TYMPHIMURIUM, corresponding to 10^3^, 10^4^ and 10^5^ log_10_CFU/cm^2^ • 2 batches of minced pork meat • 2 batches of minced beef meat
^37^	Minced pork	Spoilage	• Two independent batches of minced pork at 4, 8, and 12 °C) and under dynamic temperature conditions (i.e., periodic temperature changes from 4 to 12 °C). Sampling along 14 days (max) of storage
^38^	Chicken breast fillets	Spoilage—marination	• Breast fillets treated with five different marinades. Three different temperatures (4, 10, and 20 °C) and five marinating time intervals (1, 3, 6, and 9 h)
^39^	Fish	Spoilage	• Two independent batches with 2 replicates each of farmed whole ungutted gilthead sea bream, at 0, 4 and 8 °C
^28^	Spinach and rocket	Spoilage	• Several batches of fresh and ready-to-eat rocket and baby spinach salads, stored at 4, 8 and 12 °C, as well as at dynamic storage conditions with periodic temperature changes from 4 to 12 °C (8 h at 4 °C, 8 h at 8 °C and 8 h at 12 °C). Sampling occurred periodically for a maximum time period of approximately 11 days

Another argument supporting all the above statements relative to the introduced variance due to microorganisms’ contribution is that FTIR is used as a “gold standard” method for spoilage detection and estimation^26–28^. All the studies on this field, spoilage estimation and prediction depend on those spectra variations. As described, the data for training and testing the classifier have diversities even among the same class. In general, it is well known in data science that having samples with diversities (as the spectra in our case) in the same class and for all the classes, drives the classification system towards lower generalization error. The final trained model becomes more general by virtue of being trained on more examples, incorporating the information of the diversity that can be inherent in each class. Intuitively, having a dataset of very similar spectra increases the generalization error as if the dataset was very small; most of the measurements would be like having the same multiple times. The data used for test were extracted from the original pool of data with a random generator so as to be as unbiased as can be. So, it is apparent that the data used in this research and the way they were handled, enhance the efficiency of the developed model and thus the significance of the outcome.

From all the above it can be concluded that the developed classifier apart from achieving ideal classification scores (accuracy = 1, F1-score = 1, Sensitivity = 1, Specificity = 1, Precision = 1, MCC = 1, Informedness = 1, Markedness = 1), it is also independent of sample storage conditions in terms of time, temperature, and packaging (please refer to Table SI1 for per class statistics).

In order to further evaluate and explain the efficacy of the developed classifier, the classification probabilities statistics per class were calculated and presented in Table 3. At this point the approach for the computation of those probabilities is elaborated. The *predict_function* used from the *scikit-learn* library for SVM gives the per-class scores for each sample. When the constructor (of the classifier) option *probability* is set to *True*, class membership probability estimates (using the method *predict_proba*) are enabled. In the binary case, the probabilities are calibrated using Platt scaling whereas in the multiclass case (as herein), it is extended as per Wu et al. (2004)^29^. Briefly, given the observation *x* and the class label *y*, Wu et al. (2004)^29^ assume that the estimated pairwise class probabilities *r*_*ij*_ of *µ*_*ij*_ = *P*(*y* = *i|y* = *i or j*,* x*) are available. From the *ith* and *jth* classes of a training set, a model is obtained which for any new *x*, calculates *r*_*ij*_ as an approximation of *µ*_*ij*_. Then, using all *r*_*ij*_, the goal is to estimate *p*_*i*_ = *P*(*y* = *i|x*)*, i* = *1*,…,* k*. In their research, a method for obtaining probability estimates via an approximation solution to an identity was proposed, while the existence of a solution is guaranteed by theory in finite Markov Chains.

**Table 3:** Classification probabilities statistics per class.

	Beef	Pork	Fish	Pineapple	Rocket	Chicken	Spinach
Mean	0.97	0.98	0.98	0.97	0.91	0.84	0.93
StD	0.04	0.03	0.04	0.05	0.06	0.18	0.08
Median	0.99	0.99	0.99	0.99	0.91	0.93	0.95
Max value	0.99	1.00	1.00	1.00	1.00	0.98	1.00
Min value	0.79	0.81	0.83	0.77	0.77	0.46	0.67

**Figure 2:**
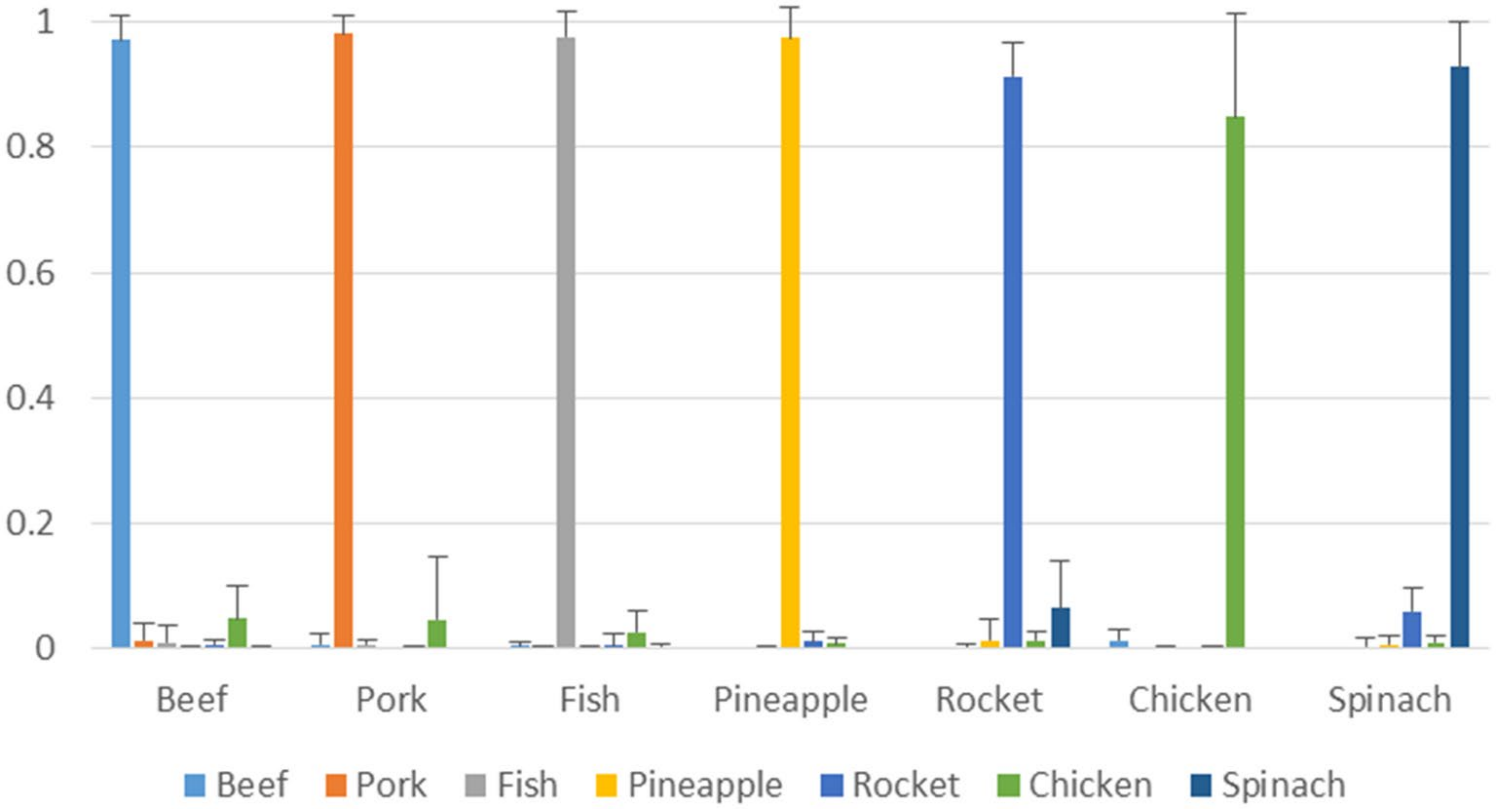
Mean class probabilities for the predictions for each class and the corresponding standard deviations.

For all the cases the mean probabilities for the classified samples is high and greater than 0.84 (with maximum standard deviation of 0.17 in the case of chicken), while the median value is > 0.91. Figure 2 shows the mean predicted probabilities for each class and the corresponding standard deviations. These high values prove the expectancy of the performance achieved, also supported but the high values of the minimum probabilities. In the case of chicken samples, the low minimum probability (0.46) accounts for just one sample whereas the next minimum value is 0.67. Those two values explain the relative large value of the standard deviation (0.17) of the probabilities. The next class probability for the chicken samples accounts to pork class (0.40), showing that pork and chicken are closely related types in terms of physiochemical properties captured by the FT-IR. It must be mentioned that pork/chicken combination is a common food fraud approach and a lot of research is undergone towards this^30–32^. It is obvious that the mean posterior probability of predicting the chicken is the worst among the others as can be noticed from the posterior probabilities provided in Table SI2, while the next highest probabilities are for pork and beef classes respectively (in all cases the highest probability is assigned to the chicken class). However it must be underlined that in no case the probability of chicken class was inferior to any other and thus there was no mismatch, as also supported by the results. Moreover, in the case of rocket and spinach classification, two types of food with high resemblance (please also refer to SI1), it can be observed that the low probability values in one result in higher values to the other, something that is expected. For instance, when for a spinach sample the probability for being spinach is 0.67, its class probability to be rocket is 0.33, and for a rocket sample for being a rocket the probability is 0.85, while for spinach is 0.14 (please refer to Table SI1 for all, per class, probabilities).

In conclusion to the aforementioned results on the generalization and efficiency of the proposed pipeline and the developed classifier, the significance of the feature selection step in tandem to the development of dedicated sensors should be highlighted. As mentioned in Methods section, the selected (41) wavenumbers were proved as the most suitable for classifying the 7 food types used here. Results like the ones presented here and others throughout the literature can drive sensor manufacturers towards building dedicated sensors for specific applications with lower cost and size that can perform optimally.

## Conclusions

Herein a uniform and global pipeline for the analysis of spectroscopic data from FT-IR towards raw food recognition has been developed and validated. As shown, the proposed workflow performed ideally on sample data exhibiting extensive variability in terms of batch, storage time, temperature, spoilage levels and packaging. This variability is reflected on the acquired spectra and proves the robustness of the method while suggesting its flawless performance. Furthermore, it can be safely concluded that FT-IR, as being a “gold standard” approach for several food safety applications and research, provides information rich data of the samples, that allows the efficient monitoring of different properties of food samples such as spoilage, quality attributes (e.g. moisture), shelf life and type of raw food (as shown herein). Apart from the efficiency shown in terms of food type classification, another critical property of the method is that its performance is invariant in terms of food storage conditions (temperature, time of storage, packaging), which makes it suitable for broad application on the detection of the raw food type. A significant property of the approach presented is that in theory it should be also efficient on different parts from a specific animal (e.g. chicken breast of wing, different parts of beef meat etc.). This can be justified via the presented results especially in the case of minced pork and/or beef, where a variety of animal parts have been used. As supported from the results, it can be concluded that the proposed workflow and the resulting classifier was able to discriminate 7 different raw food types (beef, pork, chicken, fish, rocket-salad, spinach and pineapple) with an accuracy of 100% with the data in training and testing phases exhibiting large variability in terms of batch origin and storage conditions (temperature, packaging and storage time). This enables the classifier to be robust and insensitive to random variations among the same food type. Thus the resulting classifier, as judged by its performance on a large external validation dataset, could be employed in real-world samples towards the automation of the digital food industry (e.g., food sensing devices and applications). Further research includes also the experimentation of alternative surface chemistry sensors, such as multispectral/hyperspectral imaging, that will allow us to expand towards applications such as food adulteration detection using the provided additional spatial information of the food samples.

## Methods

### Methodology

First and prior to supervised dimensionality reduction via Partial Least Squares (PLS) regression, Standard Normal Variate (SNV) normalization scheme17 and specifically under its robust version, RNV^18^ was employed to normalize the acquired spectra *S,* according to:

1 $$ {s_{i}^{snv}} = \frac{{{s_{i}} - median\left( S \right)}}{mad\left( S \right)} $$

where *s*_*i*_ is the *i*th spectrum and *s*_*i*_^*snv*^ the *i*th normalized spectrum. *MAD* stands for Median Absolute Deviation (mad)^33^; a robust variability metric of a univariate sample of quantitative data *s*_*1*_*,s*_*2*_*,…,s*_*n*_. MAD is computed as:

2 $$ mad = median\left( {s_{i} } \right) - median\left( S \right) $$

The above normalization scheme is used for data quality enhancement, reduction of the correlated information along the wavelengths of the spectra and eliminate the multiplicative noise originating from the acquisition process inherent in order to improve the downstream analysis. The same scheme for data normalization has been used in another work by our laboratory^34^.

Then the normalized data/spectra, are passed to the next step of processing, i.e. dimensionality reduction, and more specifically PLS-based supervised dimensionality reduction. In general, PLS regression^19^ is used for finding relationships between two data matrices, X and Y via a linear multivariate model. It is a popular method with extensive applications in numerous scientific fields and it’s well suited for applications where the number of variables (wavelength/wavenumbers in our case) exceeds the number of samples. PLS resembles in many ways with Principal Component Analysis^35^ (PCA) as it transforms and maps a set of possibly correlated observations to a set of linearly uncorrelated values called components and the space those components define. Some of these components can then be used as regression coefficients for the data to accomplice dimensional reduction. PLS in contrast to PCA takes into account the latent structure of not only the independent variables (predictors) but that of the dependent (responses) as well. This is done by using a training set to find the multi-dimensional variance direction from the predictors’ space, where the maximum multidimensional variance direction is explained into the space of the responses. This way, one can predict the response of new data based only on the predictors using a model created in a supervised manner.

**Figure 3:**
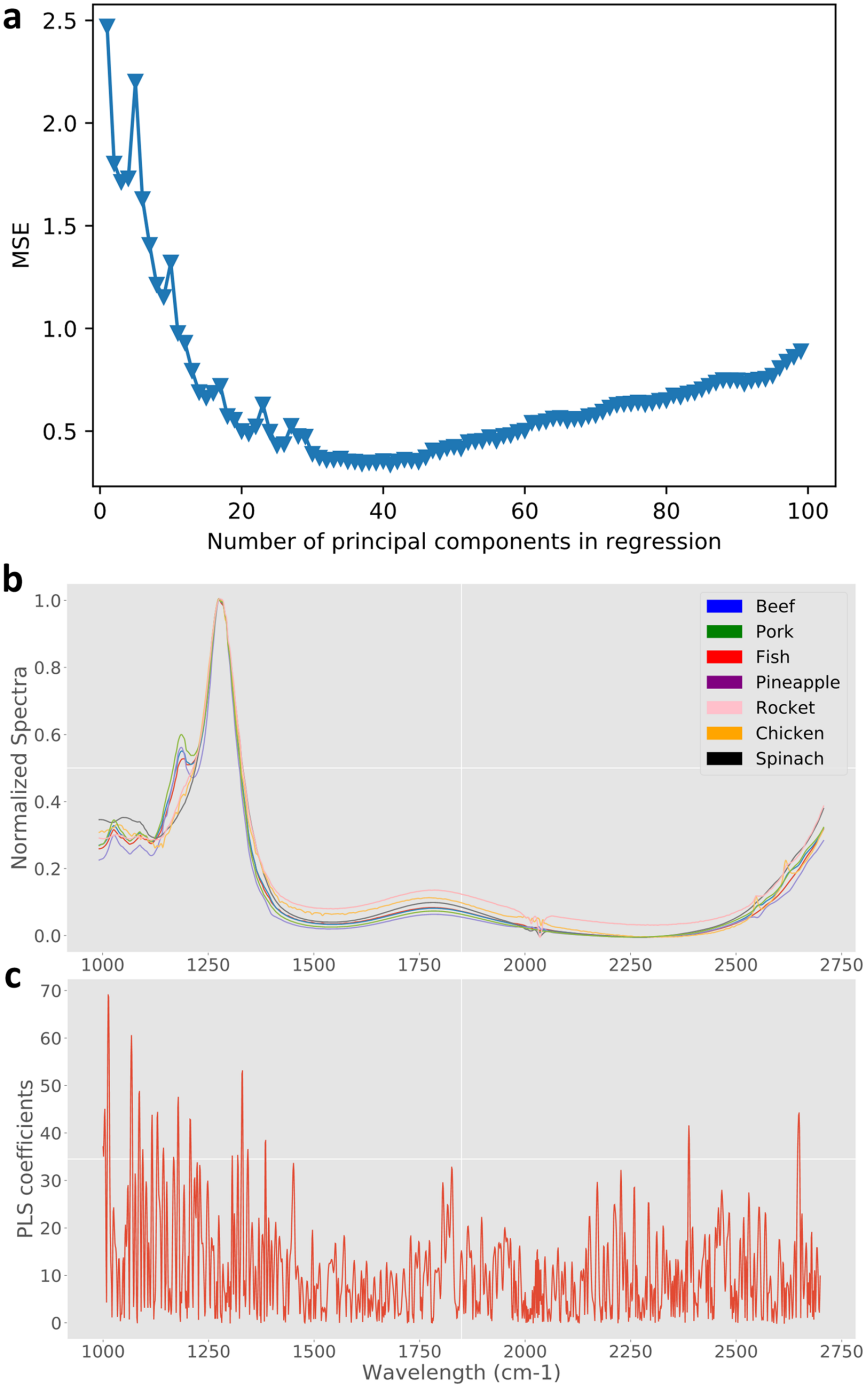
Supervised PLS dimensionality reduction overview: (**a**) mean square error vs. number of components (minimum MSE @ 41 components) across tenfold cross-validation, (**b**) sample spectra for each class type, (**c**) weights from PLS for each coefficient, i.e. wavelength.

The number of the components to “hold” in a PLS regression^19^ modeling application is critical since wrong choice can lead or avoid “over-fitting”, i.e. a model with high accuracy on the training dataset but with little to zero predictive power on new, independent samples. To overcome this issue usually some kind of model validation technique is used in order to assess how the resulting model will generalize with new untrained data, with the most popular being the cross-validation. During cross-validation the training data is split into training and validation sets based on a user-defined ratio. Then, recursively, several models are created and trained using the training set, every new one using one more component than the last. Each model is then evaluated with the validation set based on the mean square error (MSE) of the predicted versus the actual values (Fig. 3a). Intuitively, the smaller the MSE is, the better the model will perform towards the prediction of the response variable with new observations. Prediction error estimation with “unseen” data; i.e. with data that have not been used to train the model helps avoiding overfitting and also provide results under a fair comparison of different regressions exhibiting a variability on the number of covariates^36^. This way the inclusion of the minimum number of components in our model that best describe the data is reached. Herein, tenfold cross-validation was performed and the maximum number of components has been set to 100. The MSE metric for the calibration gets better and better the more components are added to the model (data not shown). On the other hand, the more complex the model, the more biased towards overfitting it gets. Thus, the cross-validation values of the MSE are important, so as to select the simpler model with the best performance. Figure 3a presents exactly those values, exhibiting a minimum in the MSE, which occurs for PC = 41. Adding more principal components, the cross validation MSE gets actually worse, meaning that the model starts to overfit and loses its generalization.

Next in the classification pipeline, is the Support Vector Machine (SVM) based modeling with the use of the data transformed into the PLS space and at the selected number of components, i.e. problem dimensionality. Support Vector Machines (SVMs) are models, created in a supervised manner, used for classification and regression analysis^20^. SVMs is a well-suited classification technique when the training data consists of large number of variables in relationship to the number of observations. In SVM every sample x that consists of n variables is treated as an n-dimensional vector. The goal is to find a surface, a hyperplane that is able to separate the vectors based on their corresponding class. There can be many hyperplanes that can classify the data. From those hyperplanes the ones that have the lowest generalization error, i.e. best predictive capability of new untrained data, need to be chosen. One common way to achieve that is by choosing the hyperplane that represents the largest “separation”, or margin, between the classes. This hyperplane has the largest distance from the closest training data point of any class. In many cases the sets to discriminate cannot be linearly separable. To overcome this, the sets can be mapped from a finite to a higher dimensional space, in an attempt to make the separation easier^37^. This is called the “kernel trick”, used for projecting and classifying the data from a higher dimensional space without ever computing the corresponding vectors in that space but rather by computing the inner products of every pair of vectors in the transformed space.

In particular, given a training set of data $\left( {x_{i} ,y_{i} } \right), i = 1, \ldots , l$ with $x_{i} \in R^{n}$ and $y \in \left[ { - 1, 1} \right]^{l}$, SVM finds the solution to the following optimization problem:

$$ min_{w,b,\xi } \frac{1}{2}w^{T} w + C\mathop \sum \limits_{i = 1}^{l} \xi_{i} $$

3 $$ {\text{subject}}\,{\text{to:}}\,y_{i} \left( {w^{T} \varphi \left( {x_{i} } \right) + b} \right) \ge 1 - \xi_{i} , \xi_{i} \ge 0 $$

The function *φ* maps the vectors *x*_*i*_ to the higher dimensional space, C is the penalty parameter of the error term and $K\left( {x_{i} ,x_{j} } \right) \equiv \varphi \left( {x_{i} } \right)^{T} \varphi \left( {x_{j} } \right)$ is the kernel function. There are many kernel functions, where the three most commonly used are:

4 $$ {\text{linear:}}\,K\left( {x_{i} ,x_{j} } \right) = x_{i}^{T} x_{i} $$

5 $$ {\text{polynomial:}}\,K\left( {x_{i} ,x_{j} } \right) = (\gamma x_{i}^{T} x_{i} + r)^{d} , \gamma > 0 $$

6 $$ {\text{radial}}\,{\text{basis}}\,{\text{function}}\,( {{\text{RBF}}}){:}\,K\left( {x_{i} ,x_{j} } \right) = e^{{ - \gamma x_{i} - x_{j}^{2} }} $$

SVM multiclass classification is implemented by the most commonly, in multiclass problems, used One-vs-the-rest (OvR) strategy, i.e. one classifier is fitted per class. As stated in the scikit-learn^38^ webpage for the multiclass algorithms, for each classifier, the class is fitted against all the other classes. The final output is the class that corresponds to the SVM with the largest margin. OvR approach is computational efficient, since only n_classes classifiers are needed, and in addition it is interpretable: each class is represented by only one classifier, thus it is possible to have access to a class by inspecting its corresponding classifier. In our case matrix X contains 1815 (training samples) normalized FTIR training samples with 1,700 variables/wavelengths each, while Y matrix is a single column matrix consisting of the class (coded as a number from 0 to 6) for each corresponding sample type. First, tenfold cross-validation was used so as to determine the minimum number of PLS components that minimize the mean squared error (please refer to Fig. 3a). The mean square error is computed by the predicted values of the class (i.e. class number) with respect to the real class number as defined by the matrix Y. In the tenfold, the training set is shuffled and then split it into ten subsets (folds). From these subsets, a single set is kept out of the training for validation and the model is trained using the rest 9 sets and repeated 10 times with a different validation set every time. This process has been employed in order to evaluate 100 different, in terms of number of components, PLS models (ranging from 1 to 100 components) (please refer to Fig. 3a). The cross-validation results for our data resulted in 41 components as the optimum number to be used. Thereafter, the model was trained using the whole of the training data, taking into account only the optimal number of components. At this point, it should be mentioned that this procedure reduced the dimensionality from 1,700 to just 41 features. Afterwards, the train data are transformed using the PLS model to get the reduced training set, which is then used as the training set for the support vector machine. In order to tune the parameters, i.e. find the optimum hyper-parameter values of SVM, grid search approach^39^ was employed. Grid search is an exhaustive search through a manually specified subset of the parameter space, combined with cross validation, in an attempt to compute the ideal kernel and parameters for the SVM classification. Herein the kernels tested were: linear, radial basis function (rbf) and polynomial, the search range for C parameter is set as [0.0001, 0.001, 0.01, 0.1, 1, 10, 100, 1000], while for the *γ* parameter [10^−6^, 10^−1^] in logarithmic scale and degree = 2, 3, 4 and 5. The outcomes for the hyper-parameters grid search resulted in choosing the linear kernel and C = 100, as the optimal classifier parameters for our data. Afterwards the SVM is trained using those optimal kernel and parameters. Finally, the same procedure is applied to the test set (described previously) after applying on them the robust SNV normalization resulted from the training set and the corresponding transformation to the same PLS space as the training set.

The SVM classification model has been evaluated on the test data, in terms of accuracy, F1-score, sensitivity, precision, specificity, Matthews’s correlation coefficient (MCC), Informedness, Markedness, in total and per class (data shown in Table SI1). In addition, the probabilities of the SVM classifier for each sample (test set) were approximated according to Platt’s scaling approach, in order to explain any misclassifications and trying to interpret the results.

### Materials and samples

The food types under consideration come from 7 different classes, namely beef^40,41^, pork^42,43^, chicken^44^, farmed whole ungutted gilhead sea bream^45^, ready-to-eat rocket^34^, baby-spinach^34^ and pineapple. The samples were all subjects from experiments and corresponding experimental setups of spoilage research, that have been previously published^34,40–45^ and the reader can refer to Table 2 and the corresponding publications for more details. In general, each food class consisted of at least 2 independent batches of samples and 2 replicates of each sample. From all these samples the corresponding spectral data have been acquired with FTIR spectroscopy, as described next. More specifically, in the case of pork, a number of samples came from Tsakanikas et al.^42^ and other additional samples came from a spoilage related experimental setup Fengou et al.^43^. Specifically, minced pork samples were prepared and packaged in food trays, placed in one styrofoam tray (duplicate samples) and wrapped with air-permeable polyethylene plastic^46^ cling film. Samples were stored at different isothermal conditions (4, 8, and 12 °C) and under dynamic conditions (periodic temperature change from 4 to 12 °C) in high precision (± 0.5 °C) programmable incubators (MIR-153, Sanyo Electric Co., Osaka, Japan) for a maximum time period of 14 days. Furthermore, two chronically independent batches have been used in order to increase samples’ variability. Concerning the pineapple samples, they were stored in their original packages at three isothermal temperatures, i.e. 4, 8 and 12 °C, and at a dynamic profile (8 h at 4 °C, 8 h at 8 °C and 8 h at 12 °C). Sampling was performed at regular time intervals depending on the storage temperature for a maximum period of 10 days. In total, four independent experimental replicates were taken into consideration resulting in 318 samples of pineapple. In summary, the total samples used in this study were 1815 training samples (400 beef, 380 pork, 300 fish, 280 pineapple, 190 rocket, 85 chicken, 180 spinach), and 240 test samples (44 beef, 52 pork, 26 fish, 38 pineapple, 29 rocket, 15 chicken, 36 spinach).

From the aforementioned description of the data used herein, it is obvious that via the high diversity of samples’ origin (different batches and in some cases even different time periods and people conducting the experiments) and state (sampling condition over a spoilage experimental setup—resulting in varying biochemical properties of the samples and thus diversity in their corresponding FTIR spectra), it was feasible to import this information into the predictive models to simulate real life conditions, since the datasets were acquired under different conditions of temperature, packaging, storage time and degree of microbiological contamination, apart from different batches. This way, it can be ensured that whatever the classification result, the model will be enough robust and generic to the input, since for different conditions the samples (within the same sample type) are degraded differently as well as their chemical profile. So, it is apparent that the evaluation scheme followed herein and more importantly, the data where the classification models were trained, are unbiased (even within the same sample type) with large variability, resulting in the development of a classifier that is robust, generic and thus reliable.

### Data acquisition—FTIR spectroscopy

The FTIR spectral data were collected using a ZnSe 45° HATR (Horizontal Attenuated Total Reflectance) crystal (PIKE Technologies, Madison, Wisconsin, USA), and an FTIR-6200 JASCO spectrometer (Jasco Corp., Tokyo, Japan). The spectra acquisition process consists of cutting a small portion from each sample and placed to the crystal plate, covered with a small piece of aluminum foil. The specific crystal works at a refractive index of 2.4 and a depth of penetration of 2.0 μm @ 1,000 cm^−1^. Then the acquired spectra were processed and collected by the Spectra Manager™ Code of Federal Regulations (CFR) software version 2 (Jasco Corp.). The corresponding wavenumber range is 4,000–400 cm^−1^, while 100 scans with a resolution of 4 cm-1 and a total integration time of 2 min were accumulated. The FTIR spectra that were used in further analyses were in the approximate wavenumber range of 2,700–1,000 cm^−1^, i.e. 1,700 wavelengths (sample features), resulted by removing the water peak starting at ~ 2,700 cm^−1^ and ignoring the range [400–1,000 cm^−1^] as it mainly represents noise.

### Implementation and performance

The whole pipeline has been implemented in Python 2.7 employing scikit-learn library^39^. The code is OS independent and require the libraries indicated in the source code and at the import instances.

### Software and data availability

The python scripts along with the data used for training and test of the system are available at zenodo, https://doi.org/10.5281/zenodo.3237542.

## Supplementary information

Supplementary file 1

Supplementary file 2
