# Supplementary material for: A machine learning workflow for raw food spectroscopic classification in a future industry
Source: Sci Rep. 2020 Jul 8;10:11212. doi: 10.1038/s41598-020-68156-2 (PMC7343812; doi:10.1038/s41598-020-68156-2)
Supplement: Supplementary file 1 [file 41598_2020_68156_MOESM1_ESM.pdf]

A machine learning workflow for raw food spectroscopic classification in a future industry

Panagiotis Tsakanikas[*,1], Apostolos Karnavas[1], Efstathios Z. Panagou[1], George-John Nychas[*,1]

Agricultural University of Athens, School of Food and Nutritional Sciences,

Department of Food Science and Human Nutrition, Laboratory of Microbiology and Biotechnology of Foods

Iera Odos 75, Athens 11855, Greece

PCA and PLS plots of the data on the space of the three first principal components

PCA

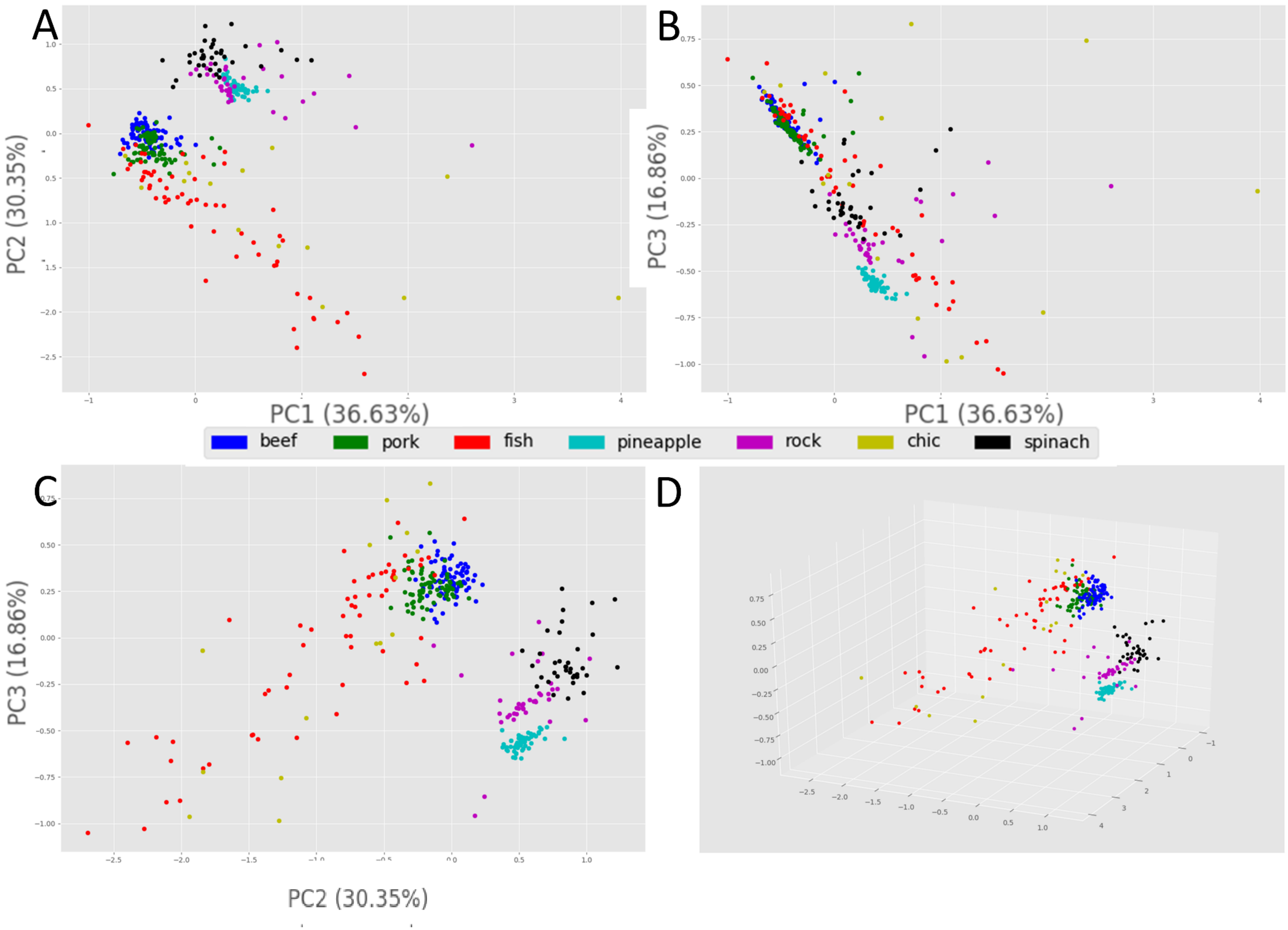

PCA plots of the normalized data: A) PC1 vs PC2, B) PC1 vs PC3, C) PC2 vs PC3 and D) 3-D plot. The percentages of the explained variances are shown on the axes.

PLS

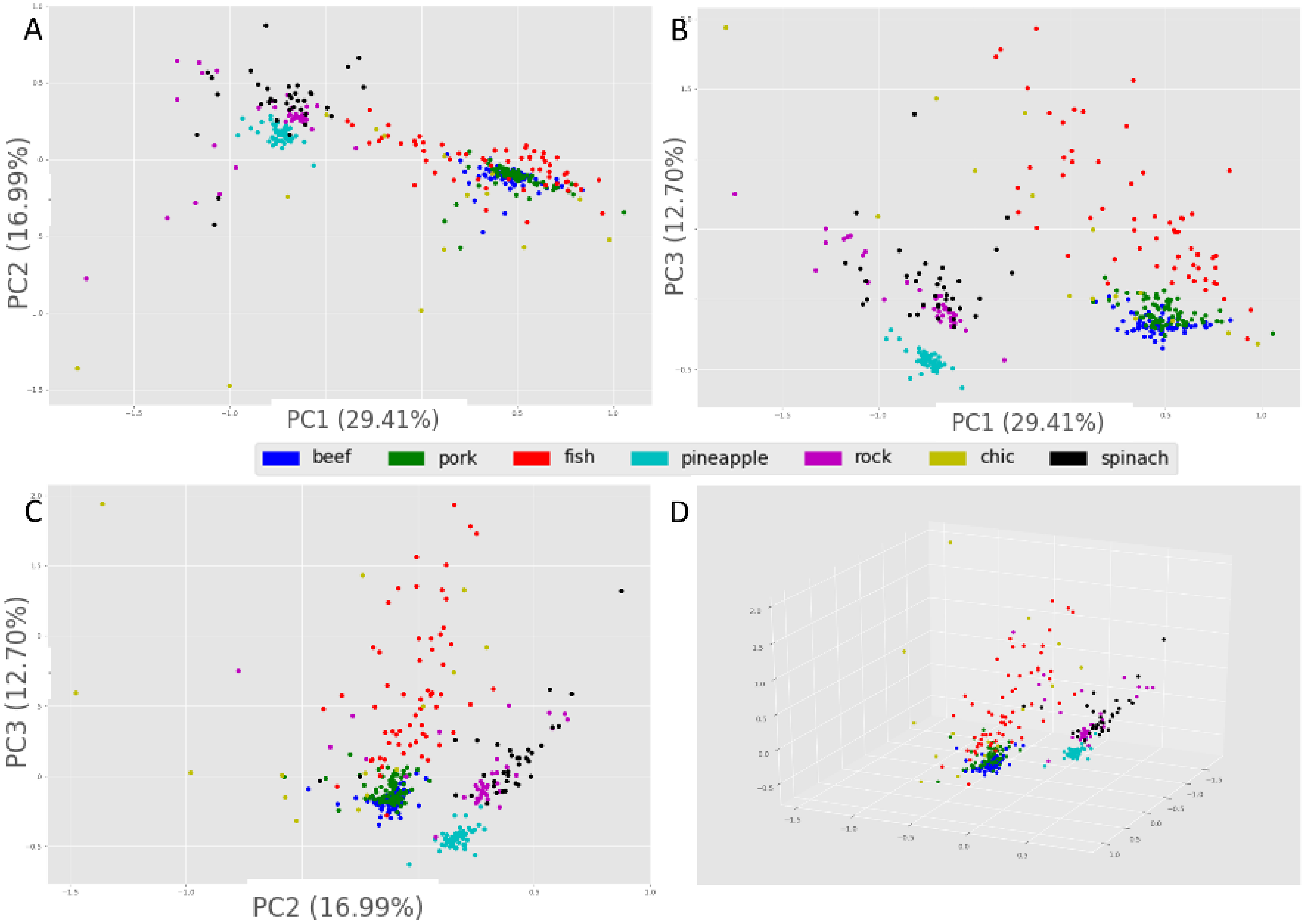

PLS plots of the normalized data: A) PC1 vs PC2, B) PC1 vs PC3, C) PC2 vs PC3 and D) 3-D plot.

FT-IR spectra (means with standard deviation) for each food category

All Raw Food samples

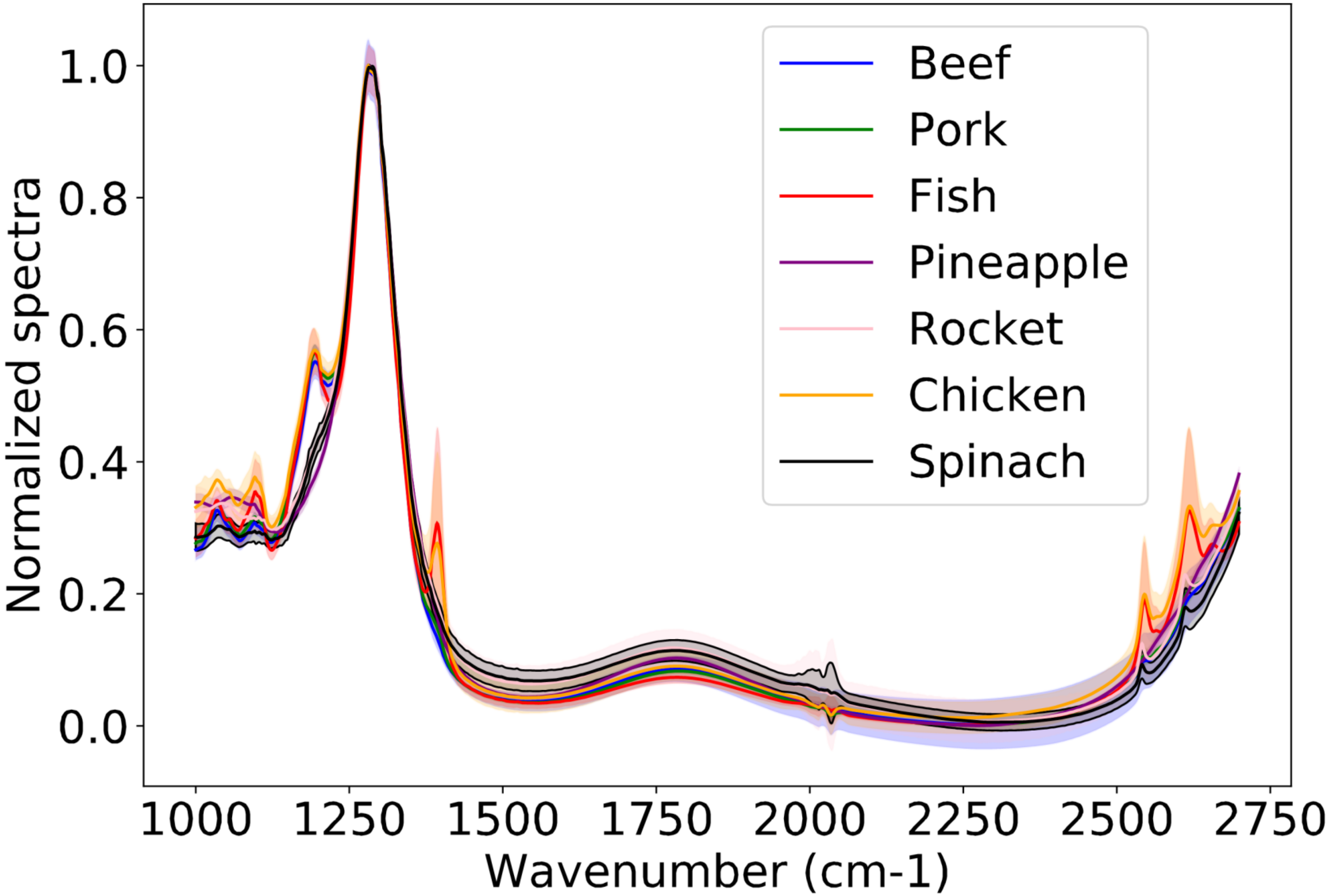

Beef samples

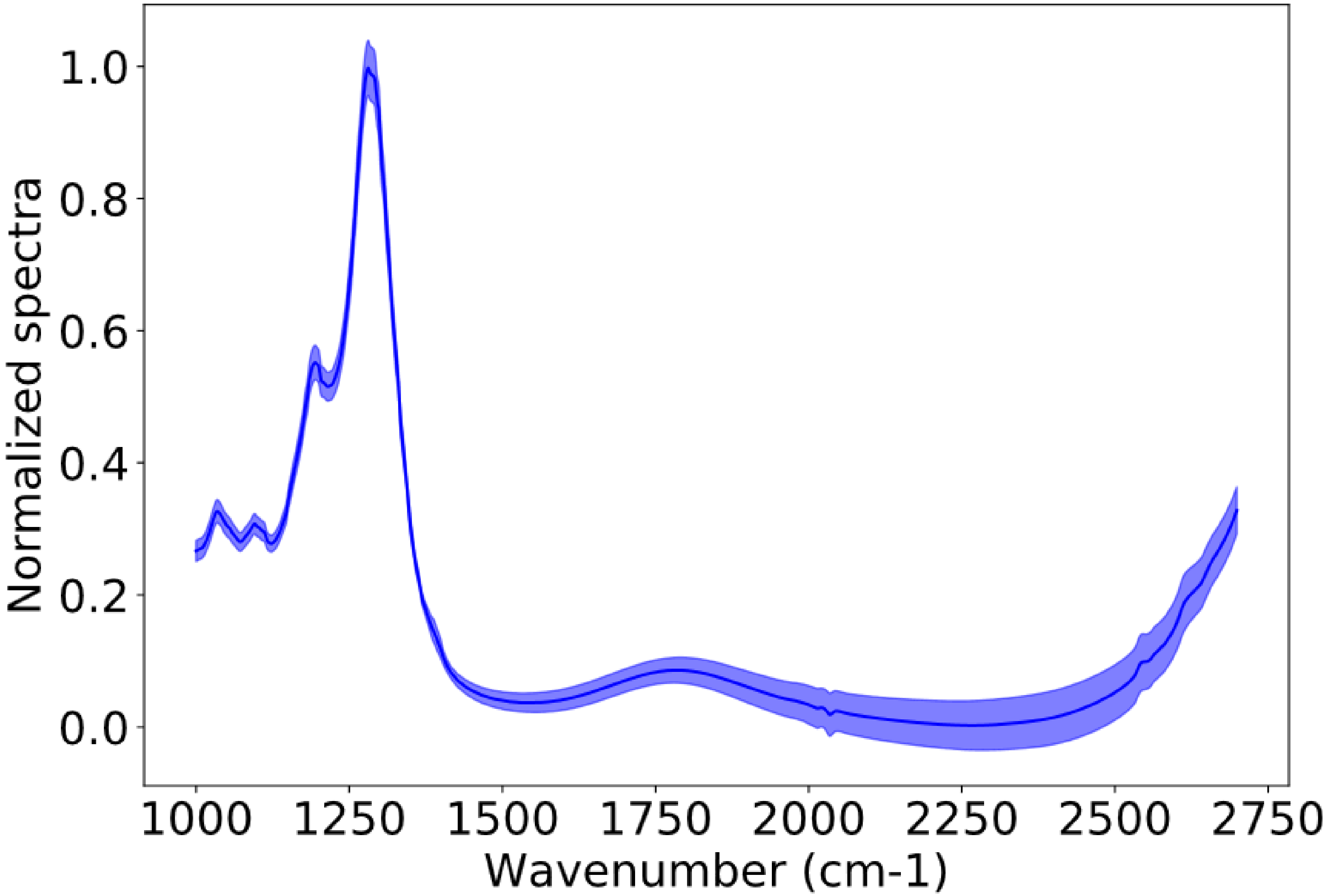

Pork samples

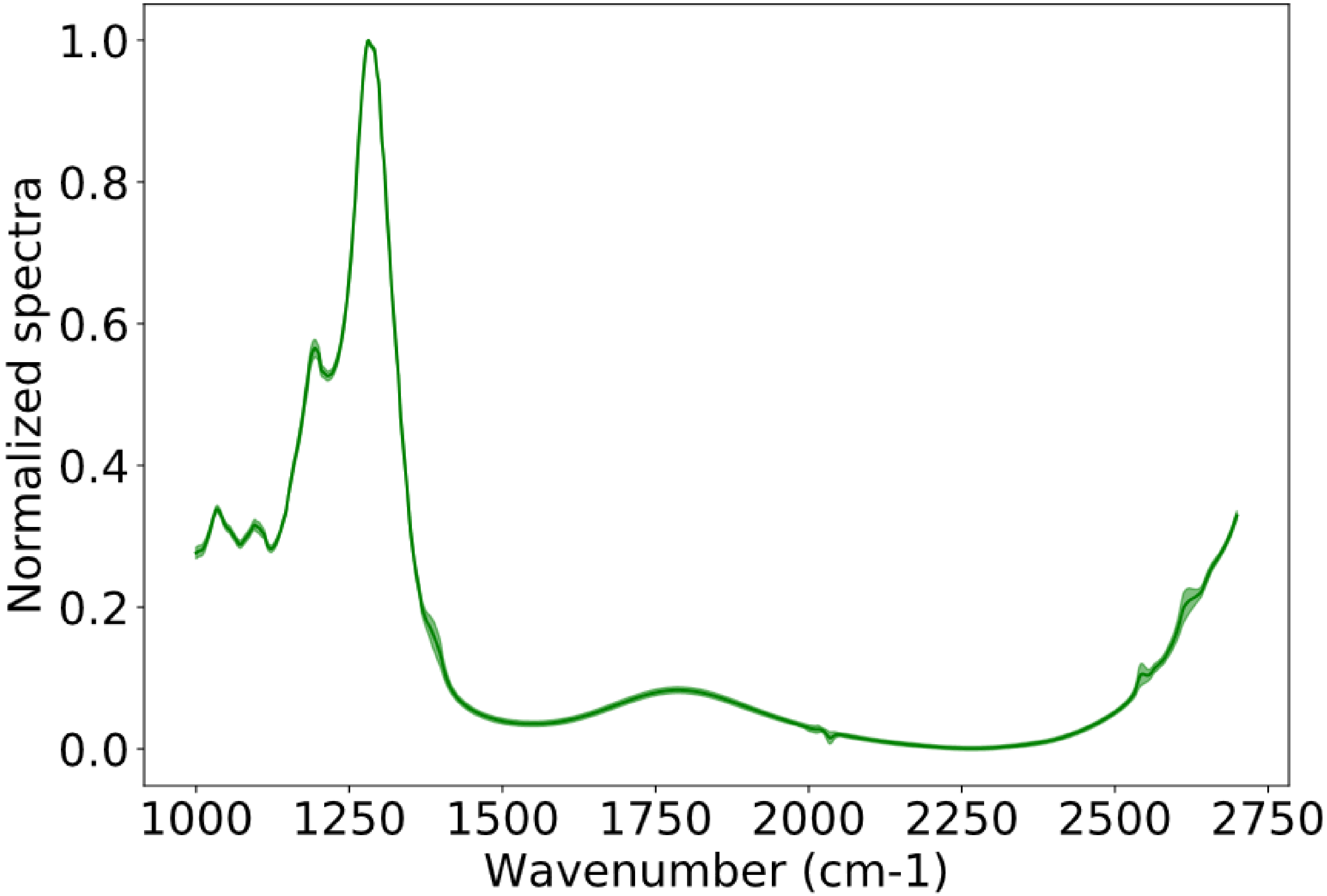

Chicken samples

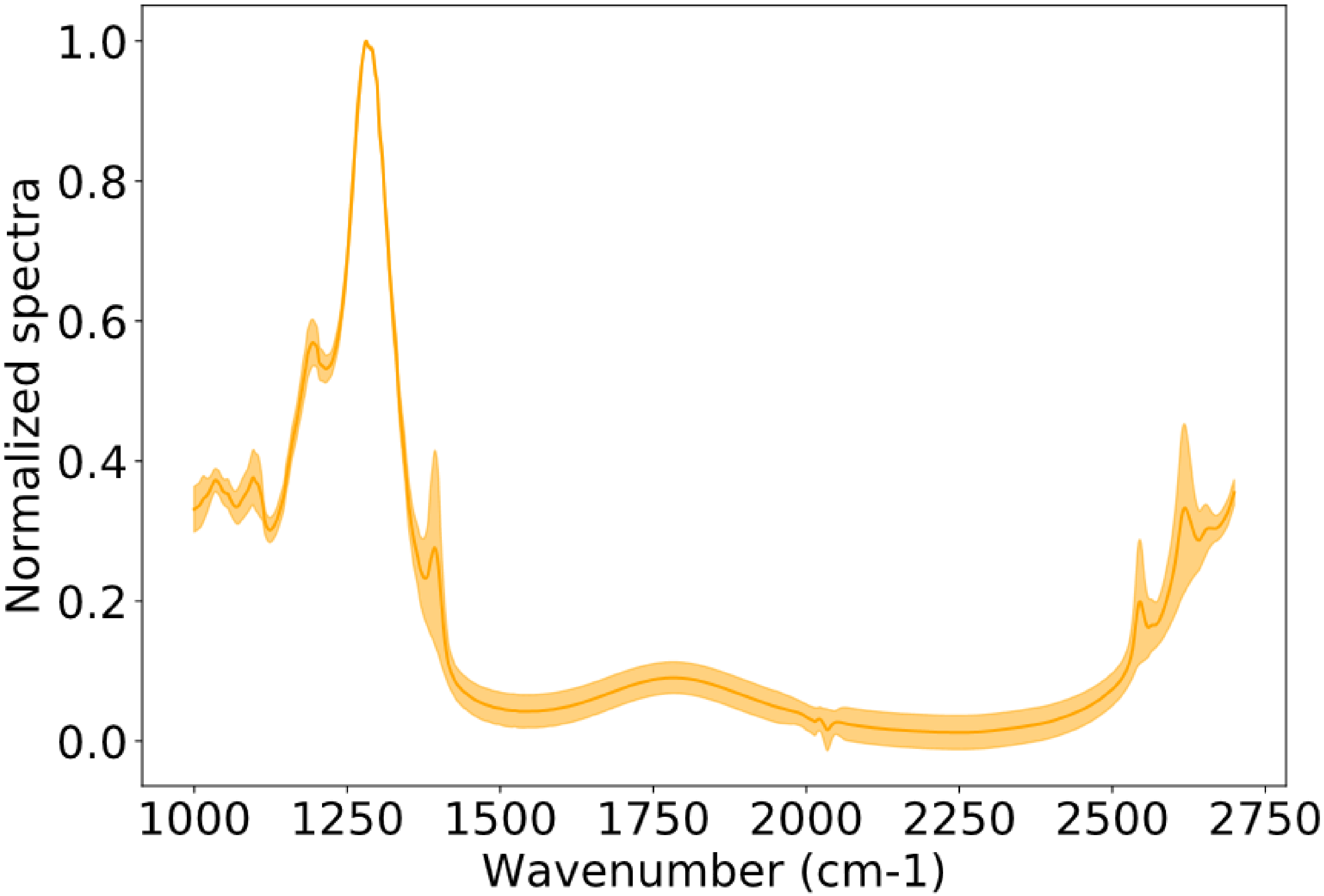

Fish samples

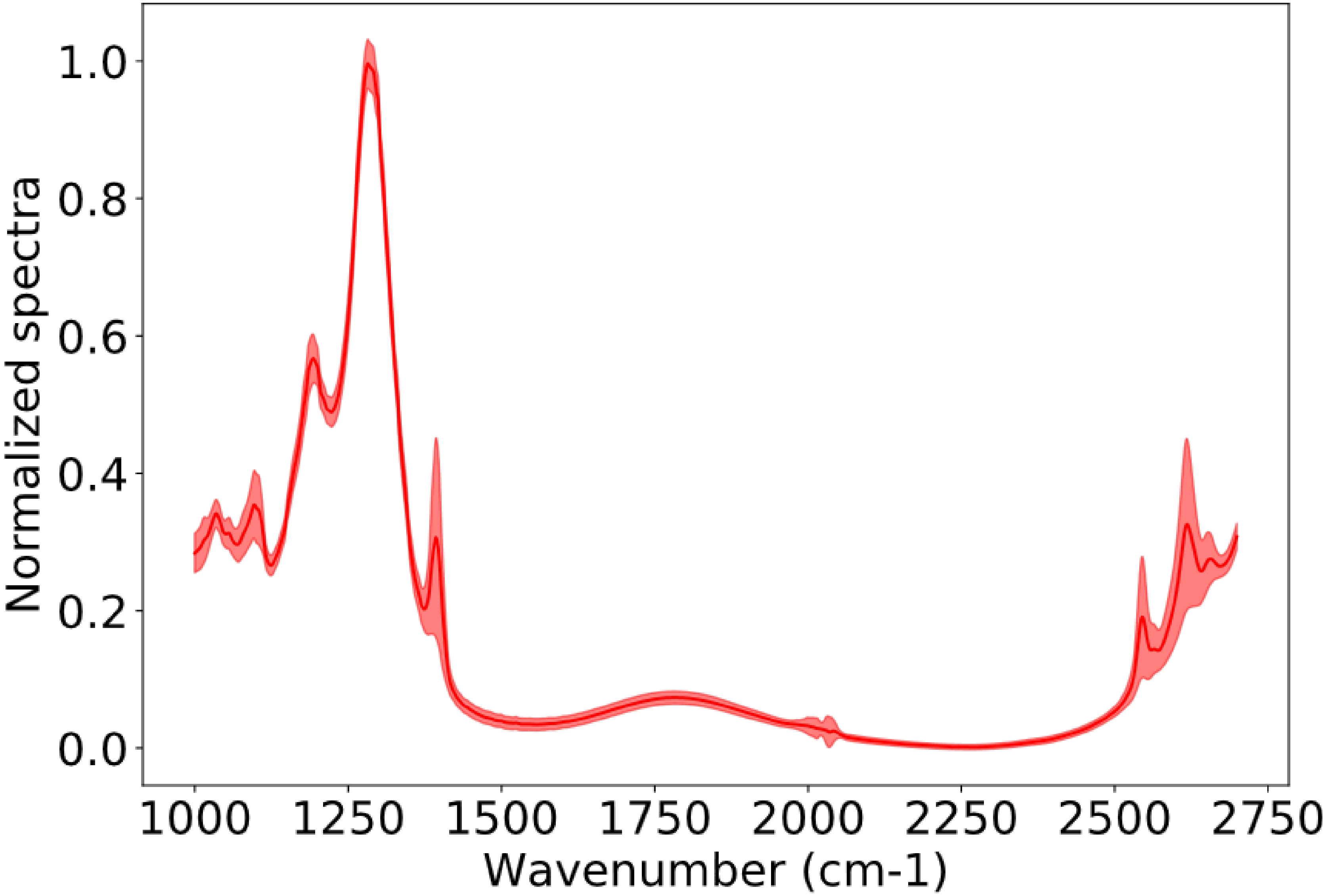

Rocket samples

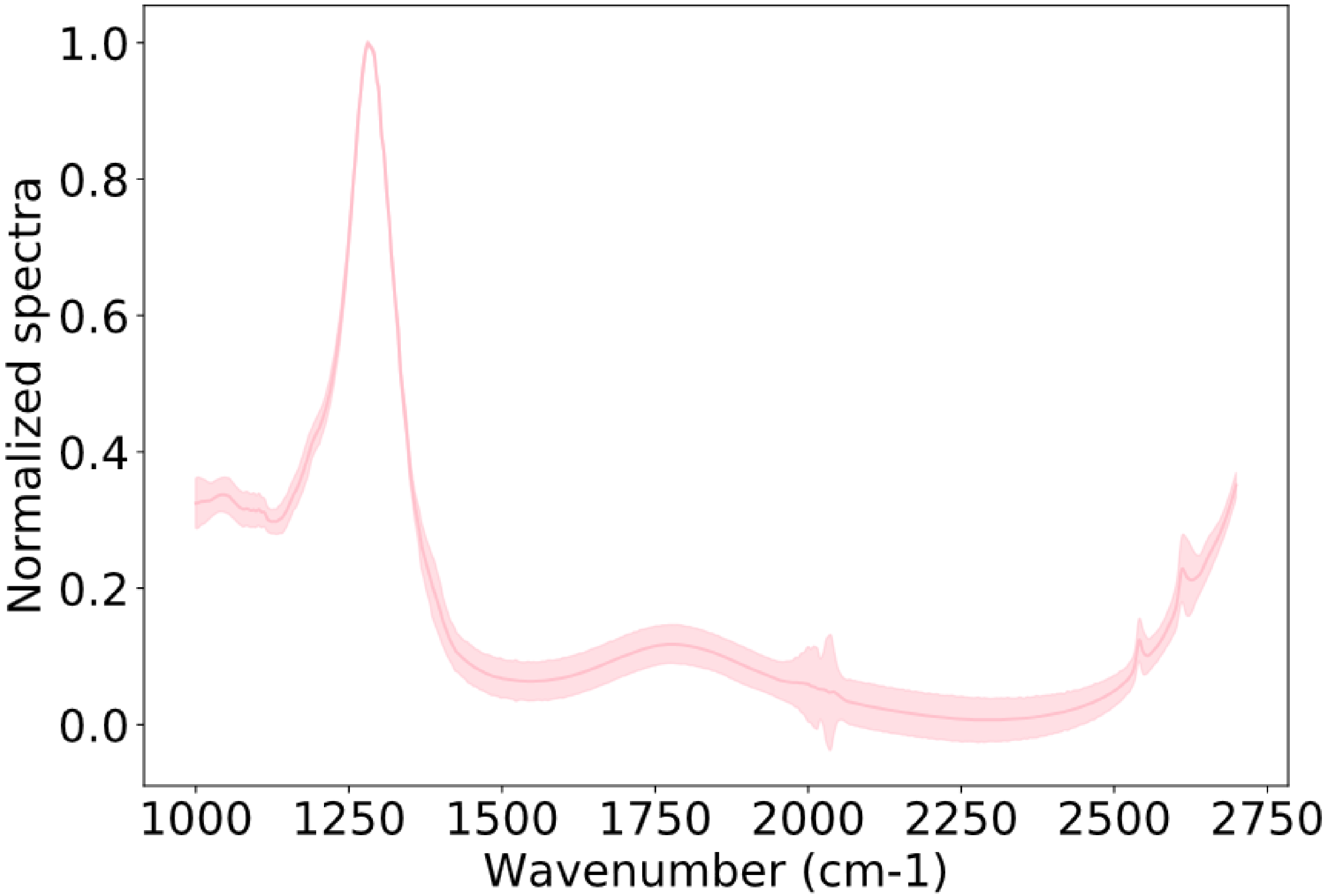

Spinach samples

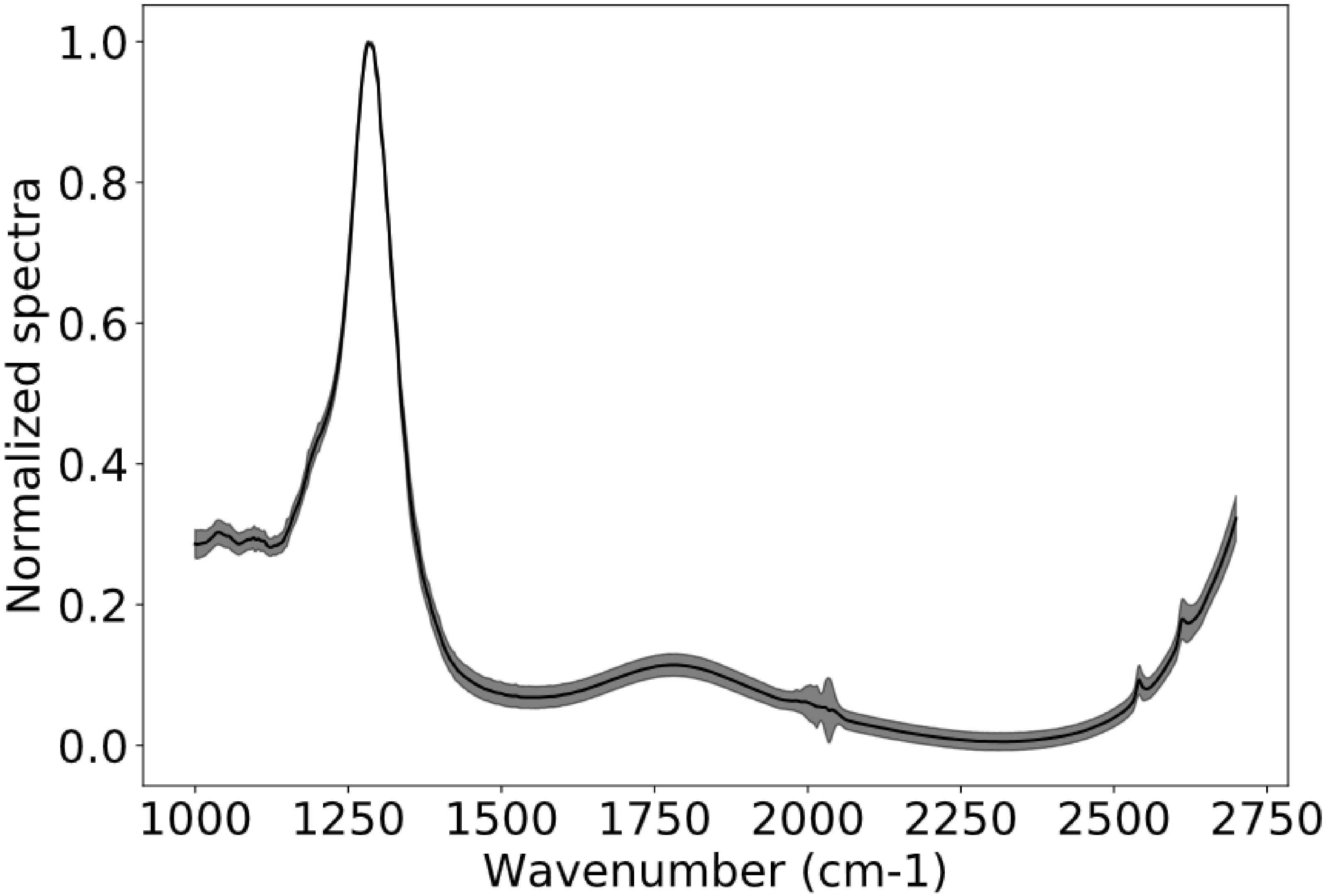

Pineapple samples

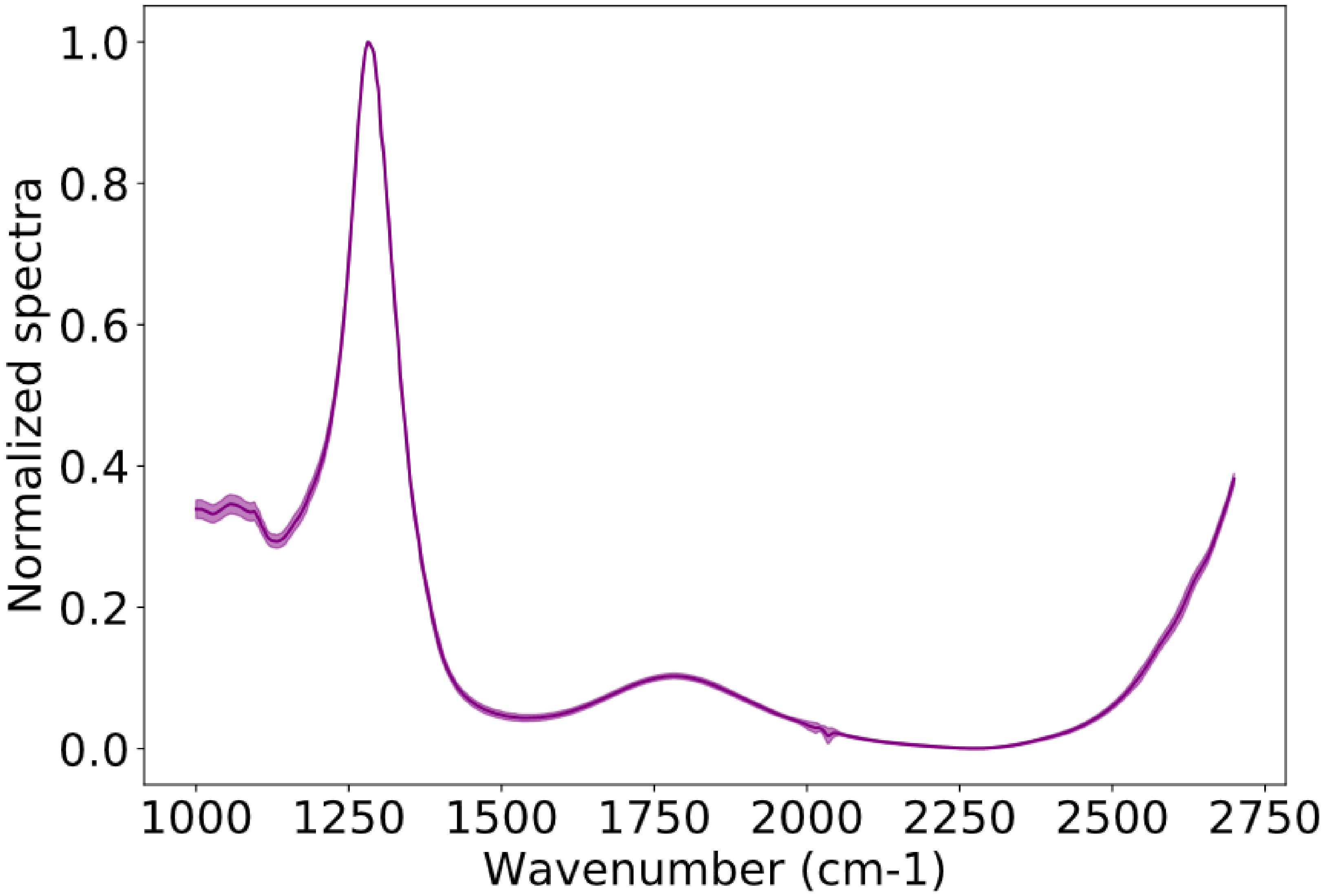

Classification evaluation measures per class

Table SI1. Classification evaluation measures per class for the test set.

	Beef	Pork	Fish	Pineapple	Rocket	Chicken	Spinach
P: condition positive	44	52	26	38	29	15	36
N: condition negative	196	188	214	202	211	225	204
Test outcome Positive	44	52	26	38	29	15	36
Test outcome Negative	196	188	214	202	211	225	204
True Positive (TP)	44	52	26	38	29	15	36
True Negative (TN)	196	188	214	202	211	225	204
False Positive (FP)	0	0	0	0	0	0	0
False Negative (FN)	0	0	0	0	0	0	0
Sensitivity (TPR)	1	1	1	1	1	1	1
Specificity (TNR)	1	1	1	1	1	1	1
Precision (PPV)	1	1	1	1	1	1	1
Negative Pred Value (NPV)	1	1	1	1	1	1	1
False-out (FPR)	0	0	0	0	0	0	0
False Discovery Rate (FDR)	0	0	0	0	0	0	0
Miss Rate (FNR)	0	0	0	0	0	0	0
Accuracy	1	1	1	1	1	1	1
Informedness	1	1	1	1	1	1	1
F1 score	1	1	1	1	1	1	1
Matthews cor coef	1	1	1	1	1	1	1
